# Coping strategies for compassion fatigue among health care professionals in intensive care units: a group concept mapping study

**DOI:** 10.3389/fpubh.2026.1798349

**Published:** 2026-03-30

**Authors:** Agnes Olander, Marie Rask, Lina Behm, Christine E. Laustsen

**Affiliations:** 1 Faculty of Health Sciences, Department of Nursing and Integrated Health Sciences, The Research Platform Collaboration for Health, Kristianstad University, Kristianstad, Sweden; 2 The Research Environment – Patient Reported Outcomes - Clinical Assessment Research and Education (PRO-CARE), Kristianstad University, Kristianstad, Sweden; 3 The Research Environment – Man–Health–Society (MHS), Kristianstad University, Kristianstad, Sweden

**Keywords:** compassion fatigue, coping strategies, group concept mapping, health care professionals, intensive care unit

## Abstract

**Introduction:**

Compassion fatigue, characterized by emotional exhaustion and reduced ability to empathize, is a well-recognized challenge among health care professionals in intensive care units. Continuous exposure to critically ill patients, ethical dilemmas and high work demands increases vulnerability to compassion fatigue and reduces compassion satisfaction. Coping strategies are essential for maintaining wellbeing and ensuring high-quality patient care. This study applies group concept mapping to identify coping strategies for preventing compassion fatigue among health care professionals in intensive care units.

**Methods:**

Group concept mapping, a participatory and mixed method, was used to collect and analyze data. Health care professionals from intensive care units in Sweden participated in data collection through brainstorming coping strategies (*n* = 27), followed by sorting (*n* = 21) and rating the strategies according to importance (*n* = 25) and use (*n* = 24). Multi-dimensional scaling and hierarchical cluster analysis were applied to generate a cluster map. Ratings of the strategies’ importance and use were analyzed.

**Results:**

Analysis of the participants’ sorting generated five clusters: (1) *Meaning making through a person-oriented perspective*; (2) *Supportive professional practice*; (3) *Peer support and empathic teamwork;* (4) *Work-life balance*; and (5) *Personal responsibility*. The cluster *Peer support and empathic teamwork* was rated highest in terms of use, while *Supportive professional practice* was rated highest in terms of importance but low in use, indicating an implementation gap. The cluster *Personal responsibility* was rated lowest in both importance and use.

**Discussion:**

The results demonstrate that coping strategies to prevent compassion fatigue operate across individual, team and organizational levels. While health care professionals value support on an organizational level, such strategies remain underutilized in current practice. Strengthening structures for ethical reflection and feedback alongside promoting support and opportunities for recovery may foster resilience and compassion satisfaction in ICU settings. Sustainable prevention of compassion fatigue requires shared responsibility between individuals and organizations, highlighting the importance of integrated approaches to staff wellbeing.

## 1 Introduction

Intensive care unit (ICU) professionals occupy a uniquely demanding role in health care, characterized by continuous meetings with patients with life-threatening conditions, rapid clinical deterioration and frequent encounters with death (1, 2). They are required to maintain constant vigilance, make split-second decisions and deliver highly specialized care under emotionally charged and ethically complex circumstances (3, 4). This sustained intensity, combined with the need to demonstrate empathy and uphold person-centered care, places ICU professionals at a distinct risk of developing compassion fatigue (CF) (2) CF is characterized by emotional exhaustion and a significantly reduced ability to feel empathy and compassion (5, 6). It has two components: burnout and secondary traumatic stress (STS) (6, 7). Burnout is characterized by emotional exhaustion, depersonalization or detachment, and low personal accomplishment (6); STS is like post-traumatic stress but results from the knowledge of traumatic events experienced by others and the consequent stress. By contrast, compassion satisfaction (CS) reflects the rewarding aspects of caregiving, offering fulfillment and a sense of purpose (6). Enhancing CS is essential, as sustained exposure to stress, trauma and suffering can erode the positive aspects of caregiving and increase the risk of CF, making ICU professionals particularly vulnerable compared with those in other health care settings (1, 2, 8). Previous studies have shown that ICU staff experience higher levels of CF and lower CS compared with nurses working in other hospital units, such as oncology or general wards (9–12).

The CF can also adversely impact the health of health care professionals. Studies have shown that those who suffer from CF are at an increased risk of long-term morbidities, such as cardiovascular disease and psychiatric illness (13, 14). It has also been found that CF can lead to individuals leaving the profession entirely (15). However, not only can CF adversely affect health care professionals but it can also negatively impact the quality of patient care (2).

It is crucial to possess compassion and empathy in the medical field, where the primary focus is providing care, comfort and, if possible, a cure for patients. Compassion and empathy entail the ability to understand and genuinely share the feelings of others (16). It is regarded as the initial step toward delivering more humane and person-centered care (2, 17), which may enhance CS (18). This underscores the importance for this group of professionals to identify effective coping strategies.

Previous studies using the Coping Strategy Indicator questionnaire have shown that ICU nurses employ various coping strategies including problem-solving, social support, and avoidance to handle and prevent CF (19–21), and there are indications that nurses who use coping strategies report higher levels of CS and experience less CF (19, 20). The need for support and effective strategies became particularly evident during the COVID-19 pandemic, when CF was increasingly recognized among intensive health care professionals. Prolonged exposure to stress, heavy workloads and emotionally demanding situations made ICU nurses especially vulnerable to CF (19). However, these challenges did not disappear with the end of the pandemic, but continue to shape the working conditions of ICU professionals today (22). At the same time, this research has primarily focused on prevalence and associated factors rather than on how context-specific coping strategies can be identified and prioritized through participatory approaches.

Against this background, CF should not be regarded as a temporary crisis phenomenon but rather, as a persistent occupational risk. This highlights the need for sustained organizational support and the implementation of evidence-based coping strategies to enhance health care professionals’ wellbeing and ensure sustainable quality of care.

While previous studies on coping strategies for CF have primarily focused on nurses (18, 20, 23), and contemporary studies continue to explore compassion fatigue primarily within this professional group (24, 25). Consequently, other health care professionals in ICUs, such as physicians and assistant nurses, remain underrepresented in coping strategy research. Including them in the research will ensure that their voices are heard.

Investigations into coping strategies to prevent CF have mainly involved cross-sectional studies (20, 23) and interview studies (18). These approaches have provided valuable insights, but rarely capture which coping strategies ICU health care professionals use to prevent compassion fatigue, or how they themselves evaluate the feasibility and appropriateness of these strategies within their organizational context. While these studies have expanded our understanding of CF and its management, they also reveal significant gaps in knowledge, particularly concerning how coping strategies are perceived and applied across different health care professional groups.

Furthermore, most existing studies were conducted before or during the COVID-19 pandemic, a period that fundamentally changed the working conditions and psychological demands in intensive care. Emerging evidence from post-pandemic studies demonstrates that burnout and emotional strain remain high among ICU health care professionals (22, 26), yet little is known about how coping strategies are currently prioritized and applied in everyday clinical practice. As health care systems have since undergone structural and organizational transformations, it is unclear whether previously identified coping strategies remain relevant or effective in the current context. This shift underscores the need to explore how ICU health care professionals utilize coping strategies to prevent CF in the post-pandemic context.

Although various coping strategies for CF have been explored, no previous studies in intensive care have applied a participatory approach. Therefore, this study uses group concept mapping (GCM) to identify and prioritize coping strategies among ICU health care professionals. Building upon this participatory foundation, the study contributes to a practical understanding of how coping strategies can be applied and sustained. By deepening our understanding of how coping strategies are perceived and implemented in today’s ICU environment, this study aims to bridge the gap between research and clinical practice. Preventing and managing CF is essential to safeguard the wellbeing of health care professionals and maintain the quality of care provided to patients in the ICU. Accordingly, this study aimed to identify coping strategies to prevent CF among health care professionals in the ICU.

## 2 Methods

### 2.1 Design

**Figure 1:**
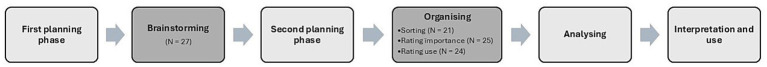
Illustration of the GCM process and the number of participants in each step.

Group concept mapping, a participatory and mixed method, was used to achieve the aim of this study. Starting with a qualitative phase followed by a quantitative phase, the method is similar to an exploratory sequential method (27). GCM involves the participants in several steps of the process; hence, it ensures that their world-view is presented in the results. The GCM process consists of the following six steps: (a) the first planning phase; (b) brainstorming; (c) the second planning phase; (d) organizing; (e) analyzing; and (f) interpretation and use (28). A web-based system, Concept System® groupwisdom™ (Concept Systems Inc., Ithaca, NY, United States), was used for data collection and analysis. A reporting guideline for GCM was followed (29). Figure 1 illustrates the process and number of participants in each step. It should be noted that GCM is a flexible method that allows for different participants to participate in different steps (30). Participation was anonymous; hence, no data can be related to specific participants or departments.

### 2.2 GCM data collection process

#### 2.2.1 The first planning phase

During the first planning phase, the aim of the study and the focus prompt were developed. A test of the focus prompt was carried out by two people who worked in an ICU. Based on their brainstorming, no changes were made to the focus prompt; however, in the introduction text for online brainstorming, it was specified to include strategies that could be used either at the workplace or in personal life. Purposive sampling (27) was used to reach participants with experience and knowledge in the focus area. The inclusion criterion was: being employed in an ICU in Sweden.

To establish contact with potential participants, contact was made with ICUs in four hospitals in northern and central Sweden, and a social media platform was used. Information about the study was emailed to the four ICUs. The operational managers at each ICU had agreed that their staff could participate in the study and forwarded information about the study and a link to the web-based system to them. Furthermore, information about the study and a link to the web-based system were posted in a social media group on Facebook, which consisted of nurses who worked in an ICU and included 867 members. Thus, in addition to purposive sampling, recruitment involved elements of network-based dissemination through managerial and professional networks, resembling aspects of snowball recruitment.

Data were collected from May 2024 to February 2025. A sample size of 20–30 participants was deemed sufficient to obtain an acceptable stress value for the statistical analysis; therefore, this sample size was strived for in each phase (31). Those who agreed to participate were asked to fill in a questionnaire about gender, age, profession, working hours per week, and length of work experience in an ICU (see Table 1).

**Table 1:** Characteristics of participants in the different steps of the data collection process.

Participant characteristics	Brainstorming *n* = 27 (%)	Sorting *n* = 21 (%)	Rating importance *n* = 25 (%)	Rating use *n* = 24 (%)
Gender				
Women	22 (81)	19 (90)	20 (80)	19 (79)
Men	5 (19)	2 (10)	5 (20)	5 (21)
Age, yrs				
18–30	1 (4)	1 (5)	3 (12)	3 (13)
31–40	8 (30)	7 (33)	9 (36)	9 (38)
41–50	7 (26)	12 (57)	9 (36)	8 (33)
51–60	11 (41)	1 (5)	4 (16)	4 (17)
Profession				
Assistant nurse	3 (11)	3 (14)	6 (24)	6 (25)
Nurse	20 (74)	15 (71)	16 (64)	15 (63)
Doctor	4 (15)	2 (10)	2 (8)	2 (8)
Manager	–	1 (5)	1 (4)	1 (4)
Working hrs/wk				
20–40	23 (85)	19 (90)	21 (84)	20 (83)
>40	4 (15)	2 (10)	4 (16)	4 (17)
Yrs working at ICU				
1–2	4 (15)	5 (24)	8 (32)	8 (33)
3–5	2 (7)	3 (14)	4 (16)	4 (17)
6–10	7 (26)	1 (5)	3 (12)	3 (13)
11–15	6 (22)	7 (33)	7 (28)	6 (25)
16–20	2 (7)	3 (14)	2 (8)	2 (8)
>20	6 (22)	2 (10)	1 (4)	1 (4)

#### 2.2.2 Brainstorming

Brainstorming was conducted via the web-based system, allowing the participants to log in at times that suited them and brainstorm on the focus prompt “A strategy used, or which can be used, to prevent CF is …”. They were guided to think about different experiences they had, and strategies that could be used, both at the workplace and in private life. The term “CF was defined in the introduction to the brainstorming phase as “*a condition where an individual feels exhausted, overwhelmed, or numb because they are being continuously exposed to the feelings and needs of others. It can occur when a person does not achieve adequate recovery or receive adequate support to cope with the emotional burden. CF can lead to difficulties in feeling compassion and commitment to the person they are caring for.*” When logging into the system, participants were able to see a list containing all brainstormed statements by other participants. A reminder to participate in the online brainstorming was sent out three times to contacts at the ICU and on social media. All together, 27 people contributed statements to the focus prompt.

#### 2.2.3 The second planning phase

The statements generated during the online brainstorming were reviewed and synthesized by the authors AO and CEL. To enable an audit trail (32) and for the authors MR and LB to conduct a quality control of the synthesis, a systematic process was implemented using Excel, arranging statements with similar meanings or content in the same row in a document. During this synthesis process, one statement per row was formulated to capture the content and meaning of all included statements, while preserving the participants’ original wording as closely as possible. Statements not related to the focus prompt (*n* = 2) were removed after consensus among all the authors.

#### 2.2.4 Organizing

During the organizing phase, we re-established contact with the ICUs at the four hospitals, as well as contacting participants through social media. The participants were asked to sort and rate the statements. First, the participants (*n* = 21) sorted the statements into groups according to how they perceived these related to each other, and labeled the groups. They were instructed to sort all the statements into groups and not to create groups based on importance or groups such as miscellanies. Then, the participants (*n* = 25) rated the statements according to importance, facilitated by the question: “*How important is the strategy described in the following statements for preventing CF*” Furthermore, the participants (*n* = 24) rated the statements according to feasibility, facilitated by the question: “*How often is the strategy suggested in the following statements used to prevent CF?*” For both ratings, they were asked to use a scale of 1–4, where 1 = not at all; 2 = a little; 3 = a lot; and 4 = very much. They were instructed to use the whole scale.

### 2.3 Analyzing

Multi-dimensional scaling (MDS) analysis and hierarchical cluster analysis were conducted on the participants’ sorting of the statements. By using the Concept System® groupwisdom™, a two-dimensional map was created by first calculating a similarity map and then using MDS analysis to place the statements on the map. Each point represents a statement, and the closer the points are to each other on the point map, the more frequently the statements have been sorted together by the participants. The lower the stress value, the better the fit between the similarity matrix and the point map (31), which in GCM studies typically falls between 0.10 and 0.35 (28). In addition, bridging values (BVs) were calculated to further examine the relation between the statements. A BV ranges from 0 to 1 and reflects the aggregate sorting by the participants. A statement with a low BV has mostly been sorted with statements nearby. When a statement has a high BV, it has mostly been sorted with statements that are placed further away on the map (28).

Afterwards, hierarchical cluster analysis was conducted, which resulted in the cluster solution. The authors AO and CEL conducted the analysis, and the preliminary results were discussed among all the authors until consensus on a cluster solution was reached. The statement with the lowest BV in the cluster can be seen as an anchor, meaning that it represents the core idea of the cluster. The aggregated BV from all the statements in a cluster is represented in the cluster’s BV. A cluster with a low BV is a more homogeneous cluster, while a cluster with a high BV is a heterogeneous cluster. Labeling of the clusters was discussed among all authors, considering the participants’ suggestions of labels, the anchor (the statement with the lowest BV in the cluster) and the content of all statements in the cluster. This process aims to give the clusters labels that reflect the clusters’ content.

To analyze the ratings, the average rating was calculated and displayed in a bivariate scatterplot, which enabled comparison of the statements’ rating in terms of importance and use. Furthermore, the average rating of all the statements in each cluster was calculated to compare differences in rating at the cluster level.

### 2.4 Interpretation and use

To validate the preliminary results and the interpretation, a member check of the results can be conducted with the participants of the study or other users with experience in the area (32). In this study, the preliminary results were presented to ICU professionals and their leaders at an ICU. This presentation led to a discussion where the professionals could relate to the cluster descriptions and the ratings of use and feasibility. They also discussed how they could use the results. The practical implications of the results will be further elaborated on in the Discussion and concluding sections.

## 3 Results

**Figure 2:**
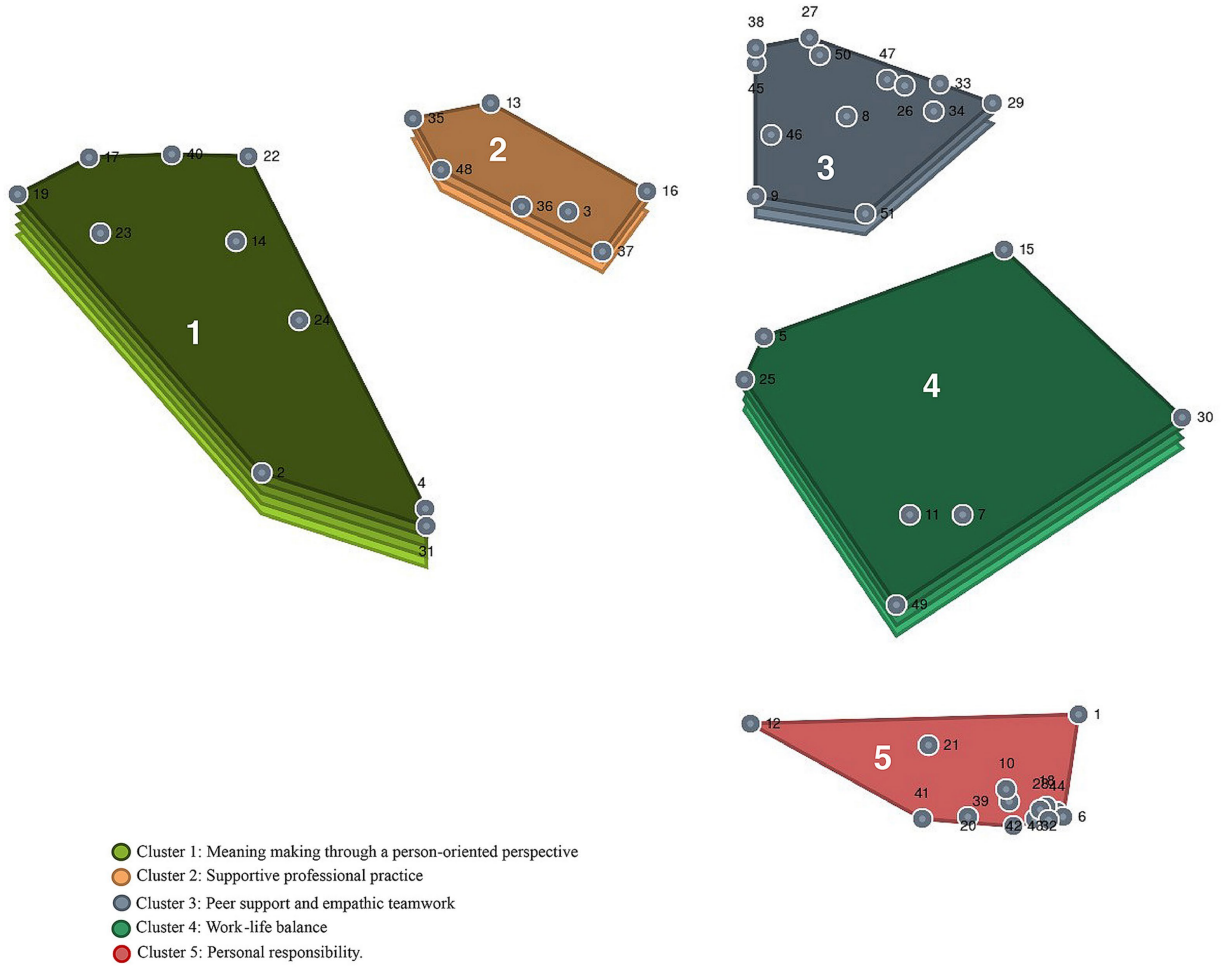
Point cluster map.

In all, 154 statements were collected during brainstorming and then synthesized into 51 statements. The statements and respective clusters, as well as BVs and average ratings, are presented in Table 2. The participants’ sorting of the statements is illustrated in the point cluster map in Figure 2. Each point illustrates a statement, and the locations of the points on the map depend on the participants’ sorting; the more often statements have been sorted together, the closer they appear on the map. The stress value from the MDS analysis was 0.20, indicating a good fit between the similarity matrix and the point map.

**Table 2:** Clusters and statements on strategies for preventing CF.

Cluster solution and statements		Bridging value^a^	Average rating of importance^b^	Average rating of use^c^
Cluster 1: Meaning making through a person-oriented perspective		0.81	2.8	2.51
2	To focus on what has gone well	1	3.2	2.55
4	To not focus on what one has not had time to do	0.81	2.76	2.42
14	To optimize medical record documentation	0.73	2.32	1.83
17	To interact with patients in a good way	0.84	3.52*	3.18
19	To use the patient’s name	0.89	2.88	3.41*
22	To receive feedback about patients who are improving	0.68*	3.2	2.23
23	To have the opportunity to care for the same person over a longer period	0.78	2.12	2.3
24	To be able to see things from different perspectives	0.8	3.08	2.68
31	To not spend too much energy on things that cannot be changed	0.84	2.88	2.64
40	To have a designated nurse in charge of nursing care	0.75	2.08	1.87
Cluster 2: Supportive professional practice		0.47	3.37	2.37
3	To plan and optimize staff allocation based on the number of patients and their care needs	0.4	3.52*	2.45
13	To have the opportunity for reflection and discussion of ethical aspects	0.53	3.36	2.32
16	To receive support from the manager	0.37*	3.52*	2.5*
35	To have the opportunity for individual reflection during working hours	0.57	3	2
36	To receive feedback from one’s employer	0.44	3.52*	2.35
37	To have the opportunity for education	0.4	3.44	2.5*
48	To have an ongoing discussion about an appropriate level of care	0.55	3.2	2.43
Cluster 3: Peer support and empathic teamwork		0.38	3.32	2.7
8	To have well-functioning teamwork	0.36	3.52	2.91
9	To have an extra resource available to provide support during periods of high care intensity	0.38	3.36	2.27
26	To feel confident to speak openly with colleagues	0.36	3.52	2.82
27	To have the opportunity to reflect and have a discussion with a colleague during working hours	0.35	3.32	2.43
29	To greet colleagues by their name	0.48	3	3
33	To have mutual respect among colleagues	0.35	3.56	2.86
34	To have good colleagues	0.38	3.64	3.23*
38	To share personal experiences of CF with colleagues	0.38	2.8	1.86
45	To exchange strategies for managing CF with colleagues	0.36	2.84	2
46	To contribute to an empathetic atmosphere at work	0.42	3.4	2.95
47	To receive collegial support in difficult situations	0.34*	3.68*	2.76
50	To be able to have discussions within the team	0.35	3.24	2.86
51	To have the opportunity to take breaks during work hours	0.47	3.28	3.1
Cluster 4: Work-life balance		0.6	3.09	2.46
5	To have reduced working hours	0.49	3.04	2.05
7	To be able to make use of wellness benefits	0.61	3.04	2.59
11	To have the opportunity to recover between shifts	0.58	3.56*	2.57
15	To have fun at work	0.62	3.32	3.24*
25	To have the opportunity to take micro-breaks during working hours	0.55	3	2.18
30	To go to after-work gatherings with colleagues	0.84	2.08	1.95
49	To be able to disconnect from work when off duty	0.48*	3.56*	2.65
Cluster 5: Personal responsibility		0.1	2.66	2.31
1	To treat oneself to something tasty to eat	0.26	2.36	2.25
6	To practice yoga	0.02	1.76	1.35
10	To read a book	0.05	1.88	1.91
12	To have balance between work, leisure activities and family life	0.51	3.52	2.52
18	To spend time with friends	0.01	3.12	2.23
20	To have a hobby	0.08	2.72	2.59
21	To get enough sleep	0.22	3.56*	2.77
28	To watch movies or a TV series	0.01	2	2.3
32	To spend time in nature, such as the forest, the sea or the garden	0*	3.04	2.73
39	To spend time with family	0.05	3.44	3.14*
41	To not have too much planned during time off	0.15	2.52	2.32
42	To have a creative hobby, such as handicrafts or playing an instrument	0.01	2.04	1.59
43	To go for a walk	0*	2.68	2.32
44	To exercise, e.g., go jogging	0.01	2.56	2.32

^a^BV: Statements marked with asterisks (*) have the lowest BV in their respective cluster.

^b^Average rating of importance: Asterisks (*) indicate the highest rated statements in each cluster regarding how important the strategy is for preventing CF.

^c^Average rating of use: Asterisks (*) indicate the highest rated statements in each cluster regarding how often the strategy is used to prevent CF. Bold value indicates the cluster name, average BV, and average ratings of importance and use within each cluster.

### 3.1 Clusters of coping strategies

The hierarchical cluster analysis resulted in a cluster map (see Figure 2) comprising five clusters, illustrating areas of strategies for preventing CF. The clusters were: Cluster 1: *Meaning making through a person-oriented perspective*; Cluster 2: *Supportive professional practice*; Cluster 3: *Peer support and empathic teamwork*; Cluster 4: *Work-life balance*; and Cluster 5: *Personal responsibility*. The clusters’ BVs are illustrated as layers of the clusters. The BV of each cluster and statement is presented in Table 2. Furthermore, Table 2 also lists the average rating value of each cluster and statement regarding importance and use.

*Cluster 1: Meaning making through a person-oriented perspective* illustrates strategies related to encounters with patients when working as a health care professional in an ICU. Strategies to prevent CF can be finding meaning through a person-oriented perspective. This involves seeing the patient the health care professional is caring for as a person, by, for example, using the person’s name and having the opportunity to care for the same person over a longer time, and receiving feedback about patients who are improving. Also, focusing on what has gone well and not spending too much energy on what cannot be changed are strategies to prevent CF, along with being able to see things from different perspectives.

The average BV for the cluster is 0.81, meaning that the statements in the cluster have often been sorted with other statements in the map, illustrating that this is a heterogeneous cluster. Statement 22, “To receive feedback about patients who are improving,” has the lowest BV (0.68) and is the anchor statement. Statement 17, “To interact with patients in a good way,” was rated highest in terms of importance (3.52) and statement 19, “To use the patient’s name,” was rated highest in terms of use (3.41).

*Cluster 2: Supportive professional practice* highlights strategies related to ICU health care professionals’ need for support and development in order to cope with and prevent CF. For example, health care professionals need to have the opportunity for education and for reflection and discussion on ethical aspects. Support from one’s manager and feedback from one’s employer are likewise useful strategies. Furthermore, the professionals saw it as a strategy to be actively involved in an ongoing discussion about the appropriate level of care, as well as ensuring that the staff allocation is planned and optimized, based on the number of patients and their care needs.

The cluster has an average BV of 0.47, meaning that it is a somewhat heterogeneous cluster and that the statements are related to other clusters in the map. The anchor statement is statement 16, “To receive support from the manager” (0.37), which was also rated highest in terms of importance (3.52), together with statement 3, “To plan and optimize staff allocation based on the number of patients and their care needs” (3.52), and statement 36, “To receive feedback from one’s employer” (3.52). Statement 16 was also rated highest in terms of use (2.5), together with statement 37, “To have the opportunity for education” (2.5). It is noticeable that Cluster 2 and its statements’ average rating were rated highest in terms of importance (3.37) but second lowest in terms of use (2.37).

*Cluster 3: Peer support and empathic teamwork* illustrates strategies to prevent CF through teamwork among co-workers in the ICU. Having well-functioning teamwork with mutual respect among colleagues is important, as is being able to have discussions within the team and receive peer support, for instance exchanging strategies for managing CF with colleagues. Moreover, contributing to an empathetic atmosphere at work and feeling confident to speak openly with colleagues were reported as strategies, including having the opportunity to reflect and have a discussion with a colleague during working hours.

The average BV for the cluster is 0.38. The statement with the lowest BV (0.34) is statement 47, “To receive collegial support in difficult situations,” which was rated highest in terms of importance (3.68). “To have good colleagues” (statement 34) was rated highest in terms of use (3.23). Overall, this cluster was rated highest with regard to use (2.7) by the ICU health care professionals.

*Cluster 4: Work-life balance* highlights strategies for preventing CF among the ICU health care professionals by balancing their work and private life. Being able to recover between shifts and disconnecting from work when off duty were reported as strategies used by the professionals. Other strategies reported to ensure work-life balance were to have reduced working hours and make use of wellness benefits; having fun at work and participating in after-work gatherings with colleagues was also reported.

The cluster has an average BV of 0.6, illustrating that it is a fairly homogeneous cluster. Statement 49, “To be able to disconnect from work when off duty,” is the statement with the lowest BV (0.48), being the anchor statement in the cluster. Statement 49, together with statement 11, “To have the opportunity to recover between shifts,” it was rated highest in terms of importance (3.56). Statement 15, “To have fun at work,” was rated highest in terms of use (3.24).

*Cluster 5: Personal responsibility* illustrates strategies that fall within the ICU health care professionals’ individual responsibilities to prevent CF. The cluster concerns achieving good integration of work, and enjoying leisure activities and family life. Several of the statements relate to different hobbies, such as handicrafts or playing an instrument, or activities in one’s spare time, such as reading a book, watching movies or a TV series. More active activities such as exercise, practicing yoga or going for a walk were also reported, as well as spending time with family or friends.

This cluster is the most homogeneous cluster on the map, with a BV of 0.1. “To go for a walk” (statement 43) and “To spend time in nature, such as the forest, the sea or the garden” (statement 32) are the anchor statements in the cluster, both with a BV of 0. However, several of the statements in the cluster have BVs close to 0. Statement 21, “To get enough sleep,” was rated highest in terms of importance (3.56) and statement 39, “To spend time with family,” was rated highest in terms of use (3.14). This cluster has the lowest average ratings in terms of both importance (2.66) and use (2.31).

### 3.2 Importance and use of coping strategies

**Figure 3:**
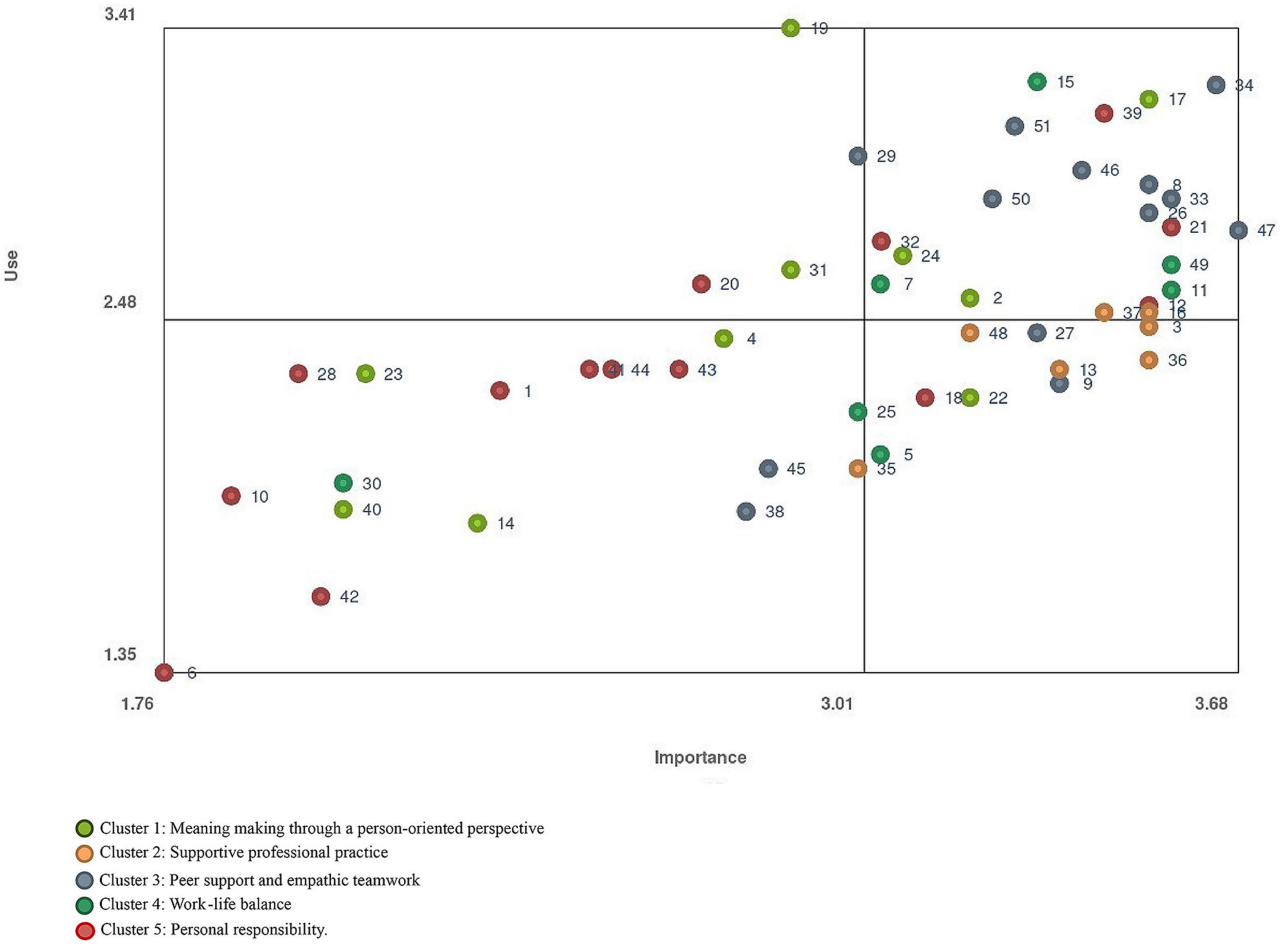
Scatterplot illustrating the relation between rating in terms of importance and use for each statement.

The relation between importance and use in the statements’ average rating is illustrated in a bivariate scatterplot (Figure 3). The colors of the points illustrate which cluster they belong to. The x-axis illustrates importance and the y-axis illustrates use. The position of the lines dividing the bivariate scatterplot into quadrants was determined by the mean values of the two ratings. There is quite a strong positive correlation in the ratings (*r* = 0.67), meaning that if a statement is rated high in terms of importance it usually is also rated high in terms of use. The statements in the upper right quadrant are rated highest in terms of both importance and frequency of use, indicating that these strategies should be prioritized for continued use in the future. The statements in the lower right quadrant are rated high in terms of importance but low in frequency of use, indicating that these strategies should be prioritized for improvement. Several statements from the cluster *Supportive professional practice* are located in this quadrant suggesting that “To have an ongoing discussion about an appropriate level of care” (statement 48), “To have the opportunity for reflection and discussion on ethical aspects” (statement 13), “To plan and optimize staff allocation based on the number of patients and their care needs” (statement 3) and “To receive feedback from one’s employer” (statement 36) are all important strategies for ICU professionals to prevent CF but are, according to the professionals’ experience, rarely used. The statements in the upper left quadrant are rated low in terms of importance but high in frequency of use, and the statements in the lower left quadrant are rated low in terms of both importance and frequency of use.

**Figure 4:**
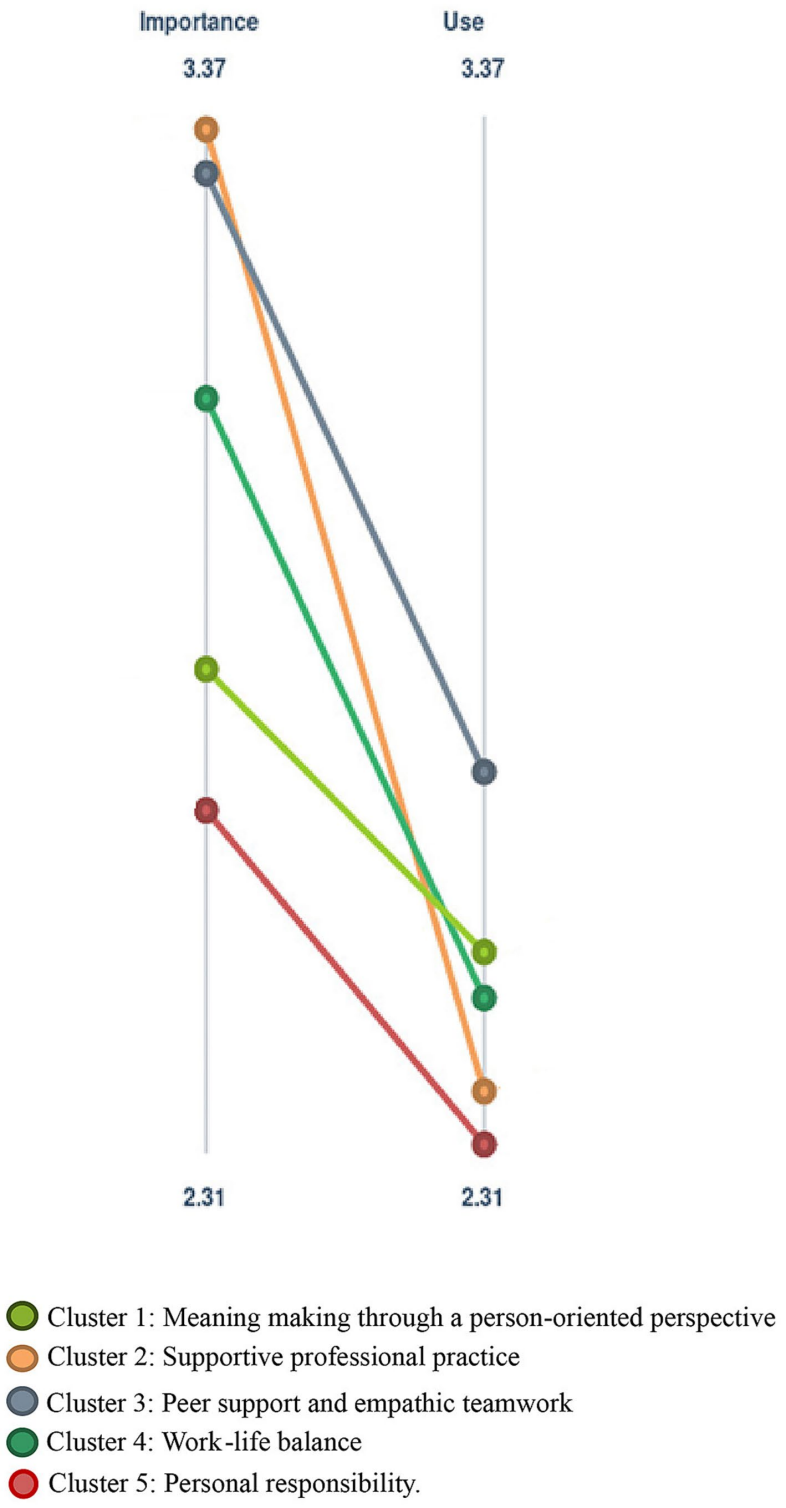
Rating at the cluster level.

Figure 4 illustrates the average rating, and differences between the average ratings, at the cluster level regarding both importance and use of strategies. Presenting the results at the cluster level, the Figure contributes to an overall understanding of how clusters, i.e., groups of related strategies, are perceived and applied. The Figure shows that *Supportive professional practice* is rated as highly important but also includes rarely used strategies for preventing CF. The cluster *Peer support and empathic teamwork* is the next highest ranked cluster in terms of importance and also the highest ranked in terms of use, implying that ICU professionals support each other as a strategy for preventing CF. The cluster *Personal responsibility* was rated lowest regarding both importance and use.

## 4 Discussion

This study aimed to identify coping strategies to prevent CF among health care professionals in intensive care units, with particular attention paid to the importance and use of the coping strategies. The results illustrate that these coping strategies are not confined to one level of practice but span individual, collegial and organizational dimensions. Some strategies are already well integrated into daily routines, while others are recognized as important but remain underutilized. Understanding how these coping strategies are valued and used provides valuable insights into both the complexity of CF and the opportunities for sustainable prevention.

The results indicate that certain areas of strategies illustrated as clusters were perceived as important and as being applied to a greater extent than others. One of the most prominent was *Peer support and empathic teamwork*, which included strategies such as having the confidence to speak openly with colleagues, finding opportunities for reflection and dialog during working hours, as well as supportive collegial relationships, sharing personal experiences, and receiving support from colleagues in challenging situations. The importance of collegial support is also highlighted in previous research. For example, Zhang et al. (24) demonstrated that nurses’ ability to cope with emotional strain and prevent CF is significantly strengthened by strong social and collegial networks. Trustworthy and supportive colleagues not only provide emotional relief but also foster a sense of belonging, mutual understanding and shared responsibility. Collegial support may therefore act as a buffer against CF. However, while collegial support provides an essential buffer against CF, it cannot compensate indefinitely for systemic shortcomings such as understaffing or limited recovery opportunities. This underscores the interdependence between micro-level strategies within teams and macro-level organizational responsibility (33).

Another area of strategies that was frequently used concerned *meaning making through a person-oriented perspective*. This included focusing on positive outcomes, refraining from dwelling on tasks that could not be completed, engaging meaningfully with patients, addressing patients by name, receiving feedback on patients’ improvements, and being able to care for the same individual over an extended period. These findings resonate strongly with the principles of person-centered care. This is noteworthy, since it has been argued that person-centered care could, under certain circumstances, increase the risk of CF (34). By contrast, Youn et al. (35) report that CS is a significant predictor of person-centered care in ICU settings. These differing outcomes suggest that person-centered care may contribute to both positive and negative emotional experiences among ICU nurses, depending on contextual or other personal factors.

A further area of strategies that emerged from the results concerned *work-life balance*. This encompassed reduced working hours, the possibility to recover between shifts, the opportunity to take micro-breaks during working hours, and the ability to disconnect from work during time off. Such measures are important, as they allow health care professionals to restore energy and maintain psychological boundaries, both of which are essential for preventing CF and sustaining CS. Other studies also highlight this fact; for example, O’Callaghan et al. (36) found that barriers to compassion among emergency nurses included time pressure and emergency volume. More recent findings support this, showing that nurses often struggle to achieve psychological detachment during breaks and that high workload undermines recovery efforts (37). These findings emphasize the practical importance of creating daily working conditions that enable rest, recovery and psychological detachment, which are fundamental elements in maintaining emotional availability in patient care. These strategies mainly concern organizational responsibility, particularly *supportive professional practice*, including ethical reflection, employer feedback, and involvement in staffing decisions. This gap between perceived value and actual use highlights a critical area for improvement, especially as several studies have identified work environment factors as key contributors to CF (38, 39). This suggests that sustainable prevention must include a long-term organizational commitment to structural support and professional development.

Despite being rated the most important area, *supportive professional practice* was rarely used in practice. This discrepancy raises questions about the organizational conditions required to translate perceived value into actual behavior. This area includes reflection on and discussion of ethical aspects, receiving support from the manager and feedback from the employer, as well as discussion about the appropriate level of care, care planning and optimizing staff allocation based on the number of patients and their care needs. Such practices form the foundation of a learning and ethically grounded work environment, yet their limited application suggests that structural or cultural barriers may impede their implementation. Activities such as debriefing and constructive feedback have been increasingly recognized as protective factors against CF (40). An ethical discussion during a debriefing session provides the tools for critical reflection in order to improve the ethical aspects of practice and receive support in one’s own reasoning (40). Moreover, the ability to reflect on complex patient care situations, particularly those involving end-of-life decisions or limited resources, has been shown to support emotional processing and reduce the risk of cumulative stress (41).

Taken together, these findings underscore the importance of integrating structured reflective practices into everyday clinical work. Regular managerial feedback and involvement in discussions on care standards and staffing allocation are vital aspects of a supportive professional practice. Allowing staff to contribute to workload planning based on patient acuity not only improves efficiency but also helps prevent moral distress arising from inadequate care due to resource limitations (42). However, the limited use of this strategy may suggest a lack of structural mechanisms to include frontline perspectives in administrative decision making. Its implementation depends heavily on ICU leadership culture. A recent study (43) among health care professionals found that leadership was strongly associated with satisfaction and burnout. Yet in many ICU settings, some of these activities are deprioritized because of operational pressures, time constraints or a hierarchical culture that does not always support open dialog (33).

The underuse of supportive strategies reflects a disconnect between staff needs and organizational provision, underscoring that CF is not only a personal challenge but also a systemic one (44). Unlike personal coping strategies such as exercising or engaging in hobbies, these strategies require active support from leadership, as well as structural planning and institutional prioritization. Their implementation depends not on individual motivation, but on organizational commitment to staff wellbeing and creating and maintaining sustainable working conditions. Therefore, professional development enabled by a supportive professional practice should be embedded in organizational culture. Structured reflection rounds, interdisciplinary case discussions, and feedback systems have shown promise in fostering moral resilience and reducing burnout (45). Interestingly, *personal responsibility*, an area of strategies that includes strategies on a personal level, such as engaging in hobbies, exercising, practicing yoga, or spending time with family and friends, was perceived by ICU health care professionals as both low in use and low in importance for preventing CF. This finding is noteworthy given that self-care, mindfulness and self-compassion are frequently identified in recent literature as essential components of CF prevention (25, 46). Poor sleep quality, low job satisfaction, extended working hours, and second-hand smoke exposure have been associated with STS among Chinese nurses (47).

Self-care strategies such as exercise were described as important coping mechanisms in the present study, alongside talking with colleagues, engaging in reflection and self-analysis, balancing work and personal life, developing supportive personal relationships, and seeking counseling if needed. Additionally, previous research has emphasized that a healthy lifestyle is a key factor in preventing the transition from temporary strain to chronic CF (48). Taken together, these findings indicate that although ICU health care professionals may perceive personal responsibility strategies as less relevant, the broader literature underscores their significance. Moreover, research suggests that both individual-focused and structural or organizational strategies have the potential to reduce burnout (49). One possible explanation for the low ranking of the cluster *Personal responsibility* may be the cultural and structural norms within health care environments, particularly in high-stress settings like ICUs. Health care professionals in the ICU often work long shifts with high emotional and physical demands, leaving little time or energy for personal activities (50). Therefore, strategies that require time outside of work, such as pursuing hobbies, exercising or socializing, may be viewed as unrealistic or inaccessible, despite their recognized benefits (51).

Additionally, the perception that managing CF is a personal responsibility may conflict with the broader systemic and organizational challenges facing health care professionals. There is a growing recognition in the literature that focusing solely on individual coping risks overlooks institutional responsibility for creating supportive work environments (49). When systemic issues such as understaffing, lack of mental health resources, or poor leadership are at play, individual strategies may feel insufficient or even burdensome (52). An individualized perception of CF is also highly controversial, as it risks placing shame and blame on the individual. When the concept was first introduced in the 1990s, CF was often viewed as a sign of personal weakness or a lack of professional resilience (53). This contributed to feelings of guilt, shame and inadequacy among health care professionals, fostering a culture of silence in which emotional distress was rarely discussed openly (51). However, in more recent years, the understanding of CF has shifted. Contemporary research emphasizes that CF should not be seen as a personal failure but as a shared responsibility between individuals, leadership and organizations (2).

While this broader understanding underscores the importance of organizational support, it does not diminish the relevance of individual coping efforts. Interestingly, some strategies within the cluster *Personal responsibility*, such as walking or spending time with loved ones, are widely supported as effective means of stress relief (54), yet they may not be perceived as formal or structured enough to be considered legitimate strategies for preventing CF. This could reflect a gap in how self-care is communicated and prioritized within health care settings. Education and leadership play a critical role in legitimizing personal coping efforts and encouraging staff to view self-care as a professional responsibility (55).

### 4.1 Methodological considerations

By using GCM as a method, this study generated a cluster map illustrating participants’ empirically co-created understanding of their real-world practice (28). A strength of this study is the use of a participatory and mixed method, which enabled us to capture the ICU professionals’ views through both qualitative and quantitative data collection. The participatory nature of GCM also represents a methodological strength of this study. Previous research on compassion fatigue has largely relied on cross-sectional surveys or interview designs, whereas the present approach enabled ICU professionals to actively define and prioritize coping strategies within their own organizational context. This approach may enhance the contextual relevance and potential applicability of the findings, particularly in the post-pandemic ICU environment where working conditions continue to evolve.

The GCM is a reliable and valid method (31) that integrates data collection and analysis and involves the participant in several steps of the research process. We contacted ICUs in four hospitals in northern and central Sweden and used a social media platform with 867 members to reach potential participants, and managed to include 27 participants in one of the steps. ICU staff are a group of professionals who are already experiencing a high workload, and participating in a GCM study is time-consuming; therefore, we greatly appreciate that the participants took the time to contribute to this study. A stress value of 0.20 was reached, which is an acceptable stress value in a GCM study (31). Also, GCM as a method is flexible (30) and allows for different participants to take part in different steps. Participation was made anonymous to encourage as many people as possible to take part and to allow them to feel safe and express their views freely. This consideration also means that it is unclear whether some participants took part in several of the steps, as this could not be tracked.

## 5 Conclusion

In conclusion, several strategies that can be applied in clinical practice to prevent CF have emerged from this study. The study presents an illustrative map of areas of strategies. Employing a participatory and mixed method the study shows variation in how these strategies are valued and used to ensure that both the experience and the knowledge of the ICU professionals themselves are presented. The strategies operate on different levels, both within the organization and at the individual level. Change requires more than personal responsibility, as organizational structures, leadership engagement and cultural values play a decisive role in supporting staff wellbeing. At the same time, individual coping strategies remain an important resource and should be integrated into broader health interventions. Ultimately, responsibility lies with both the individual and the organization, and the most sustainable approach is achieved when these two work together. Future research should therefore explore how health care organizations can legitimize and support individual coping efforts as part of their overall strategies to prevent CF. This includes examining how leadership, training, and workplace policies can facilitate a balance between personal accountability and organizational responsibility.

## Data Availability

The raw data supporting the conclusions of this article will be made available by the authors, without undue reservation.

## References

[1] Elkonin D van der Vyver L. Positive and negative emotional responses to work-related trauma of intensive care nurses in private health care facilities. Health SA Gesondheid. (2011) 16:1–8. doi: 10.4102/hsag.v16i1.436

[2] van Mol MM Kompanje EJ Benoit DD Bakker J Nijkamp MD. The prevalence of compassion fatigue and burnout among healthcare professionals in intensive care units: a systematic review. PLoS One. (2015) 10:e0136955. doi: 10.1371/journal.pone.0136955, 26322644 PMC4554995

[3] Teixeira C Ribeiro O Fonseca AM Carvalho AS. Ethical decision making in intensive care units: a burnout risk factor? Results from a multicentre study conducted with physicians and nurses. J Med Ethics. (2014) 40:97–103. doi: 10.1136/medethics-2012-100619, 23408707

[4] Todaro-Franceschi V. Critical care nurses' perceptions of preparedness and ability to care for the dying and their professional quality of life. Dimens Crit Care Nurs. (2013) 32:184–90. doi: 10.1097/DCC.0b013e31829980af, 23759913

[5] Figley CR Kleber RJ. "Beyond the “victim”: secondary traumatic stress". In: Figley CR, editor. Beyond Trauma: Cultural and Societal Dynamics. Plenum Series on Stress and Coping. New York, NY, US: Plenum Press (1995). p. 75–98.

[6] Stamm B. The Concise ProQOL Manual. 2nd ed. Pocatello, ID: ProQOL org (2010).

[7] Jenkins B Warren NA. Concept analysis: compassion fatigue and effects upon critical care nurses. Crit Care Nurs Q. (2012) 35:388–95. doi: 10.1097/CNQ.0b013e318268fe09, 22948373

[8] Kerlin MP McPeake J Mikkelsen ME. Burnout and joy in the profession of critical care medicine. Crit Care. (2020) 24:98. doi: 10.1186/s13054-020-2784-z, 32204724 PMC7092567

[9] Abo Elmagd MH Khamis HM Ali HYS. Determinant of work-related stress among nursing staff: a systematic review. Tanta Scientif Nurs J. (2024) 33:310–26. doi: 10.21608/tsnj.2024.351239

[10] Tsegaw S Getachew Y Tegegne B. Determinants of work-related stress among nurses working in private and public hospitals in Dessie City, 2021: comparative cross-sectional study. Psychol Res Behav Manag. (2022) 15:1823–35. doi: 10.2147/PRBM.S372882, 35923164 PMC9342705

[11] Al-Majid S Carlson N Kiyohara M Faith M Rakovski C. Assessing the degree of compassion satisfaction and compassion fatigue among critical care, oncology, and charge nurses. J Nurs Adm. (2018) 48:310–5. doi: 10.1097/NNA.0000000000000620, 29794595

[12] Özan A Polat H. Determination of compassion and compassion fatigue in intensive care nurses. SAGE Open Nurs. (2024) 10:23779608241247395. doi: 10.1177/23779608241247395, 38654971 PMC11036912

[13] John A Bouillon-Minois JB Bagheri R Pélissier C Charbotel B Llorca PM . The influence of burnout on cardiovascular disease: a systematic review and meta-analysis. Front Psych. (2024) 15:1326745. doi: 10.3389/fpsyt.2024.1326745, 38439796 PMC10909938

[14] Abo Shereda HM Alqhtani SS Alyami AH Alghamdi HM Ahmed MIO Alsalah NA . Exploring the relationship between compassion fatigue, stigma, and moral distress among psychiatric nurses: a structural equation modeling study. BMC Nurs. (2025) 24:163. doi: 10.1186/s12912-025-02802-w, 39939960 PMC11823185

[15] Sawatzky JA Enns CL Legare C. Identifying the key predictors for retention in critical care nurses. J Adv Nurs. (2015) 71:2315–25. doi: 10.1111/jan.12701, 26037809

[16] Jeffrey D. Empathy, sympathy and compassion in healthcare: is there a problem? Is there a difference? Does it matter? J R Soc Med. (2016) 109:446–52. doi: 10.1177/0141076816680120, 27923897 PMC5154411

[17] Jeffrey D. Clarifying empathy: the first step to more humane clinical care. Br J Gen Pract. (2016) 66:e143–5. doi: 10.3399/bjgp16X683761, 26823264 PMC4723213

[18] Jakimowicz S Perry L Lewis J. Insights on compassion and patient-centred nursing in intensive care: a constructivist grounded theory. J Clin Nurs. (2018) 27:1599–611. doi: 10.1111/jocn.14231, 29266484

[19] Abou Hashish EA Ghanem Atalla AD. The relationship between coping strategies, compassion satisfaction, and compassion fatigue during the COVID-19 pandemic. SAGE Open Nurs. (2023) 9:23779608231160463. doi: 10.1177/23779608231160463, 36908330 PMC9998409

[20] Al Barmawi MA Subih M Salameh O Sayyah Yousef Sayyah N Shoqirat N Abdel-Azeez Eid Abu Jebbeh R. Coping strategies as moderating factors to compassion fatigue among critical care nurses. Brain Behav. (2019) 9:e01264. doi: 10.1002/brb3.1264, 30884198 PMC6456805

[21] Ames M Salmond E Holly C Kamienski M. Strategies that reduce compassion fatigue and increase compassion satisfaction in nurses: a systematic review protocol. JBI Database System Rev Implement Rep. (2017) 15:1800–4. doi: 10.11124/JBISRIR-2016-003142, 28708743

[22] Liu H Li X Zhao L Zhang Y Huang D Li T . Burnout among intensive care physicians and nurses in the post-pandemic era in China: a national cross-sectional study. BMJ Open. (2025) 15:e099192. doi: 10.1136/bmjopen-2025-099192, 40846337 PMC12374664

[23] Jakimowicz S Perry L Lewis J. Compassion satisfaction and fatigue: a cross-sectional survey of Australian intensive care nurses. Aust Crit Care. (2018) 31:396–405. doi: 10.1016/j.aucc.2017.10.003, 29153827

[24] Zhang J Wang X Chen O Li J Li Y Chen Y . Social support, empathy and compassion fatigue among clinical nurses: structural equation modeling. BMC Nurs. (2023) 22:425. doi: 10.1186/s12912-023-01565-6, 37957600 PMC10644455

[25] Othman SY Hassan NI Mohamed AM. Effectiveness of mindfulness-based interventions on burnout and self-compassion among critical care nurses caring for patients with COVID-19: a quasi-experimental study. BMC Nurs. (2023) 22:305. doi: 10.1186/s12912-023-01466-8, 37674145 PMC10481566

[26] Yin J Zhao L Zhang N Xia H. Understanding the interplay of compassion fatigue and moral resilience on moral distress in ICU nurses: a cross-sectional study. Front Public Health. (2024) 12:1402532. doi: 10.3389/fpubh.2024.1402532 39529716 PMC11550935

[27] Hanson WE Creswell JW Clark VLP Petska KS Creswell JD. Mixed methods research designs in counseling psychology. J Couns Psychol. (2005) 52:224–35. doi: 10.1037/0022-0167.52.2.224

[28] Kane M Rosas S. Conversations about Group Concept Mapping: Applications, Examples, and Enhancements. Thousand Oaks, California: SAGE Publications, Inc.; (2018). Available online at: https://methods.sagepub.com/book/mono/conversations-about-group-concept-mapping/toc (Accessed April 02, 2025).

[29] Pantha S Jones M Gray R. Development of a reporting guideline for Trochim’s concept mapping. Methods Protocol. (2025) 8:24. doi: 10.3390/mps8020024, 40126242 PMC11932253

[30] Trochim WM McLinden D. Introduction to a special issue on concept mapping. Eval Program Plann. (2017) 60:166–75. doi: 10.1016/j.evalprogplan.2016.10.006, 27780609

[31] Rosas SR Kane M. Quality and rigor of the concept mapping methodology: a pooled study analysis. Eval Program Plann. (2012) 35:236–45. doi: 10.1016/j.evalprogplan.2011.10.003, 22221889

[32] Lincoln YS Guba EG. Naturalistic Inquiry. Newbury Park (CA): Sage Publications (1985).

[33] Kok N Hoedemaekers C Fuchs M Cornet AD Ewalds E Hom H . The interplay between organizational culture and burnout among ICU professionals: a cross-sectional multicenter study. J Crit Care. (2025) 85:154981. doi: 10.1016/j.jcrc.2024.154981, 39608045

[34] Summer Meranius M Holmström IK Håkansson J Breitholtz A Moniri F Skogevall S . Paradoxes of person-centred care: a discussion paper. Nurs Open. (2020) 7:1321–9. doi: 10.1002/nop2.520, 32802352 PMC7424463

[35] Youn H Lee M Jang SJ. Person-centred care among intensive care unit nurses: a cross-sectional study. Intensive Crit Care Nurs. (2022) 73:103293. doi: 10.1016/j.iccn.2022.103293, 35871960

[36] O’Callaghan EL Lam L Cant R Moss C. Compassion satisfaction and compassion fatigue in Australian emergency nurses: a descriptive cross-sectional study. Int Emerg Nurs. (2020) 48:100785. doi: 10.1016/j.ienj.2019.06.008, 31331839

[37] Sagherian K McNeely C Cho H Steege LM. Nurses' rest breaks and fatigue: the roles of psychological detachment and workload. West J Nurs Res. (2023) 45:885–93. doi: 10.1177/01939459231189787, 37621023

[38] Nolte AG Downing C Temane A Hastings-Tolsma M. Compassion fatigue in nurses: a metasynthesis. J Clin Nurs. (2017) 26:4364–78. doi: 10.1111/jocn.13766, 28231623

[39] Tuna R Eskin Bacaksiz F Kahraman B. Compassion satisfaction, compassion fatigue, burnout, working environments, and musculoskeletal disorders among nurses. Perspect Psychiatr Care. (2022) 58:2321–9. doi: 10.1111/ppc.13063, 35285954

[40] Patole S Pawale D Rath C. Interventions for compassion fatigue in healthcare providers-a systematic review of randomised controlled trials. Healthcare. (2024) 12:171. doi: 10.3390/healthcare12020171, 38255060 PMC10815881

[41] Rushton CH. Transforming moral suffering by cultivating moral resilience and ethical practice. Am J Crit Care. (2023) 32:238–48. doi: 10.4037/ajcc2023207, 37391375

[42] Andersson M Fredholm A Nordin A Engström Å. Moral distress, health and intention to leave: critical care nurses' perceptions during COVID-19 pandemic. SAGE Open Nurs. (2023) 9:23779608231169218. doi: 10.1177/23779608231169218, 37089200 PMC10116007

[43] Dyrbye LN Major-Elechi B Hays JT Fraser CH Buskirk SJ West CP. Relationship between organizational leadership and health care employee burnout and satisfaction. Mayo Clin Proc. (2020) 95:698–708. doi: 10.1016/j.mayocp.2019.10.041 32247343

[44] Garnett A Hui L Oleynikov C Boamah S. Compassion fatigue in healthcare providers: a scoping review. BMC Health Serv Res. (2023) 23:1336. doi: 10.1186/s12913-023-10356-3, 38041097 PMC10693134

[45] Dzeng E Colaianni A Roland M Levine D Kelly MP Barclay S . Moral distress amongst American physician trainees regarding futile treatments at the end of life: a qualitative study. J Gen Intern Med. (2016) 31:93–9. doi: 10.1007/s11606-015-3505-1, 26391029 PMC4700021

[46] Wei J Yun Z Zhang Y Liang Y Hu Z Gao C . Mindful self-care and compassion fatigue in nurses: the chain mediating roles of resilience and professional identity. J Nurs Manag. (2025) 2025:8572654. doi: 10.1155/jonm/8572654, 40223877 PMC11918527

[47] Wang J Okoli CTC He H Feng F Li J Zhuang L . Factors associated with compassion satisfaction, burnout, and secondary traumatic stress among Chinese nurses in tertiary hospitals: a cross-sectional study. Int J Nurs Stud. (2020) 102:103472. doi: 10.1016/j.ijnurstu.2019.103472, 31810017

[48] Gustafsson T Hemberg J. Compassion fatigue as bruises in the soul: a qualitative study on nurses. Nurs Ethics. (2022) 29:157–70. doi: 10.1177/09697330211003215, 34282669 PMC8866753

[49] West CP Dyrbye LN Erwin PJ Shanafelt TD. Interventions to prevent and reduce physician burnout: a systematic review and meta-analysis. Lancet. (2016) 388:2272–81. doi: 10.1016/S0140-6736(16)31279-X, 27692469

[50] Billiau L Bolliger L Clays E Eeckloo K Ketels M. Flemish critical care nurses' experiences regarding the influence of work-related demands on their health: a descriptive interpretive qualitative study. BMC Nurs. (2024) 23:387. doi: 10.1186/s12912-024-02032-6, 38844928 PMC11155134

[51] Peters E. Compassion fatigue in nursing: a concept analysis. Nurs Forum. (2018) 53:466–80. doi: 10.1111/nuf.12274, 29962010

[52] Cocker F Joss N. Compassion fatigue among healthcare, emergency and community service workers: a systematic review. Int J Environ Res Public Health. (2016) 13:618. doi: 10.3390/ijerph13060618, 27338436 PMC4924075

[53] Joinson C. Coping with compassion fatigue. Nursing. (1992) 22:116–8-9, 20. 1570090

[54] Kriakous SA Elliott KA Lamers C Owen R. The effectiveness of mindfulness-based stress reduction on the psychological functioning of healthcare professionals: a systematic review. Mindfulness. (2021) 12:1–28. doi: 10.1007/s12671-020-01500-9, 32989406 PMC7511255

[55] Smart D English A James J Wilson M Daratha KB Childers B . Compassion fatigue and satisfaction: a cross-sectional survey among US healthcare workers. Nurs Health Sci. (2014) 16:3–10. doi: 10.1111/nhs.12068 23663318

[56] World Medical Association. Declaration of Helsinki: ethical principles for medical research involving human subjects. JAMA. (2013) 310:2191–4. doi: 10.1001/jama.2013.281053 24141714

